# Inferring Relationship of Blood Metabolic Changes and Average Daily Gain With Feed Conversion Efficiency in Murrah Heifers: Machine Learning Approach

**DOI:** 10.3389/fvets.2020.00518

**Published:** 2020-09-02

**Authors:** Poonam Sikka, Abhigyan Nath, Shyam Sundar Paul, Jerome Andonissamy, Dwijesh Chandra Mishra, Atmakuri Ramakrishna Rao, Ashok Kumar Balhara, Krishna Kumar Chaturvedi, Keerti Kumar Yadav, Sunesh Balhara

**Affiliations:** ^1^Animal Biochemistry, Division of Genetics and Breeding, Central Institute for Research on Buffaloes (ICAR), Hisar, India; ^2^Department of Biochemistry, Pt. Jawahar Lal Nehru Memorial Medical College, Pt. Deendayal Upadhyay Memorial Health Sciences and Ayush University of Chhatisgarh, Raipur, India; ^3^Poultry Nutrition, Directorate of Poultry Research (DPR), ICAR, Hyderabad, India; ^4^Indian Agricultural Statistics Research Institute, Indian Council of Agricultural Research, New Delhi, India; ^5^Department of Bioinfromatics, School of Earth, Biological and Environmental Sciences, Central University of South Bihar, Patna, India

**Keywords:** buffalo, blood, feed conversion efficiency, partial least square regression, prediction models

## Abstract

Machine learning algorithms were employed for predicting the feed conversion efficiency (FCE), using the blood parameters and average daily gain (ADG) as predictor variables in buffalo heifers. It was observed that isotonic regression outperformed other machine learning algorithms used in study. Further, we also achieved the best performance evaluation metrics model with additive regression as the meta learner and isotonic regression as the base learner on 10-fold cross-validation and leaving-one-out cross-validation tests. Further, we created three separate partial least square regression (PLSR) models using all 14 parameters of blood and ADG as independent (explanatory) variables and FCE as the dependent variable, to understand the interactions of blood parameters, ADG with FCE each by inclusion of all FCE values (i), only higher FCE values (negative RFI) (ii), and inclusion of only lower FCE (positive RFI) values (iii). The PLSR model including only the higher FCE values was concluded the best, based on performance evaluation metrics as compared to PLSR models developed by inclusion of the lower FCE values and all types of FCE values. IGF1 and its interactions with the other blood parameters were found highly influential for higher FCE measures. The strength of the estimated interaction effects of the blood parameter in relation to FCE may facilitate understanding of intricate dynamics of blood parameters for growth.

## Introduction

Feed efficiency is a multifactorial functional trait reflecting the energy balance of a particular animal, which determines its overall productivity. Feed cost constitutes 70% of total input of production system profits; thus, improvement in feed utilization capacity of an animal would be very profitable (1). Expensive feed costs for milk/beef producers can be minimized by increasing feed efficiency. Beef cattle, utilizing feed efficiently, showed substantially curtailed feed consumption for comparable productive performance in contemporary animals (2). Residual feed intake (RFI) has been an accepted measure of feed utilization efficiency of animals, which defines the differences in actual and expected feed intake due to the different background energy requirements of different animals (3). Feed-efficient animal displays lower RFI, an attribute inscribed with moderate heritability of 0.15 and repeatability as 0.53 (4) and is also being used in selection programs (5). It is having limited use in industry due to its time-consuming monitoring and heavy capital investment, consequently emphasizing on the need to explore alternate approach to infer feed efficiency, as blood parameters.

The knowledge of different sources of variation that cause physiological differences among animals in terms of feed efficiency, mainly residual feed intake (RFI), is still incomplete. Variations in blood parameters and metabolic characteristics reflect appreciably a part of total feed efficiency variation in animals (6, 7). A thorough study of all possible processes related to this variation, if it does not lead to an efficient early selection, at least would be useful for deducing genotypes selectively for RFI/FCE. Blood metabolic markers associated with feed conversion efficiency were earlier used to enhance profitability (7, 8) of yearling beef bulls (9) and crossbred heifers (10), wherein the level of FCE was extrapolated on the scale of energy substrates as blood metabolite(s), i.e., glucose, triglycerides, urea, creatine phosphokinase as protein metabolite, total plasma protein, and aspartate aminotransferase, which in turn are influenced by the hypothalamus–pituitary–adrenal axis (11, 12). Several types of potential proxies for RFI, using energy metabolism (13), hepatic mitochondrial function (14), and visceral organ metabolism (15), have been identified to monitor feed efficiency in other species. Change in urea level has been associated with RFI (16), which is attributed to the rate of degradation or synthesis of protein (17), reflecting liver function and metabolic activity of the digestive tract while generating almost 40 to 50% of the total energy channeling 1.45% in animal body weight (18, 19).

An insight into the relationship of various blood parameters with ADG with FCE can shed light on physiological dynamics underlying the metabolic changes, using machine learning approaches (20–22). The biological closeness between feed efficiency and the animal's ability to convert feed nitrogen (**N**) into animal protein, i.e., N-use efficiency or N partitioning and protein turnover across individuals (23), has been used for predicting RFI in growing cattle due to the difference in rates of amino acid transamination (24).

Studies in Indian *Bubalus bubalis* to this effect are scanty. Exploring indirect markers as discriminatory change in blood attributes would be useful to frame predictive models to establish genetic markers for optimization of multifunctional complex traits as FCE. It will help in selecting efficient buffalo aptly christened “Black Gold” (25, 26), which contribute more than 22% toward worldwide demand of milk, meat, and hide (27). The mathematical model enabling the user to explore the relationship between nutrition (glucose, insulin, and IGF1 system) and reproduction is recently developed (28) for cattle as an early attempt toward developing *in silico* feeding strategies, which may reduce animal experiments eventually.

The present study is an attempt to deduce the intricate relationship between the changing dynamics of circulatory metabolites and the level of feed, utilizing efficiency in order to find out proxy indicators other than RFI and developing best-fit models to predict feed efficiency by machine learning. Blood parameters and ADG remained as predictor variables and FCE as the response variable to obtain models. Least square means were used to develop partial least square regression (PLSR) models for FCE predictions.

## Materials and Methods

### Ethical Statement

All animal experiments were performed under permission and review of the Institutional Animal Ethics Committee (IAEC) (Reg. No. 406/GO/RBI/L/01/CPCSEA). Experimental heifers (*n* = 42) used in the present study were selected at ICAR-Central Institute for Research on Buffaloes Hisar, Haryana, Govt. of India, buffalo farm to determine the levels of variation in RFI over animals under study of more than 100 days. RFI was determined as the difference in actual and predicted dry matter intake (DMI).

### Animals and Samples

Forty-two growing Murrah female buffalo calves (initial weight, 155 ± 4.6 kg; initial age, between 9 to 11 months) were utilized for the study. The buffaloes were vaccinated and treated to eliminate external and internal parasites before initiation of the study. The buffaloes were fed individually on diets comprising *ad lib* (allowing residues approximately at 10% of total daily dry matter intake) green Jowar (*Sorghum vulgare*) fodder and a fixed quantity of concentrate mixtures (50% of expected dry matter intake of individual animal). The diet was formulated to meet nutrient requirements as per buffalo feeding standards developed by Paul and Lal (29).

The quantities of offered fodder and concentrate were adjusted at fortnightly intervals depending on dry matter intake of the preceding fortnight. The residual fodder and feed were removed, weighed, and sampled for dry matter (DM) estimation before offering the next day's concentrate allowance. The DM of offered and residue fodder samples was estimated on a daily basis. The offered and residue samples of feeds and fodders were pooled at monthly intervals for chemical analysis (Table 1). Daily concentrate and fodder DM intake were recorded for each calf. Body weight changes were recorded every 2 weeks. The feeding trial continued for 96 days. One digestibility trial (comprising a 6-day collection period) involving four animals was conducted after about 65 d of feeding trial to ascertain the nutritive value of diet. The offered feeds and residues of the previous day were recorded; samples of both were collected daily and pooled throughout the experimental periods per animal for analysis. The feces voided was immediately collected and placed in covered bins animal-wise. The amounts of feces voided daily were weighed and thoroughly mixed in a pail, and an aliquot (1 g per kg fresh feces) was mixed with 15 ml of 20% H_2_SO_4_ and kept for N estimation. Another portion of the aliquot (30 g per kg (minimum of 100 g) fresh feces) was kept for drying at 70°C in a hot air oven for the estimation of dry matter and other proximate composition. Representative samples of feed offered, residues left, and feces voided were analyzed to determine nutrient digestibility. Feed and fecal samples were analyzed for dry matter (proc. # 930.15), ash (proc. # 942.05), crude protein (proc. # 988.05), and fat (proc. # 920.39) by procedures of AOAC (1990).

**Table 1:** Chemical composition of feeds^a^ (g/kg).

	**Concentrate mixture^b^**	**Jowar fodder**
Organic matter	865	943
Crude protein	241.5	60.7
Ether extract	42.9	29.9
Crude fibee	94.5	322

a *Values represent hexaplicate assays of each material*.

b *Ingredient composition of concentrate mixture: maize grain, 175 g/kg; barley grain, 175 g/kg; wheat bran, 270 g/kg; mustard cake, 200 g/kg; cotton seed cake, 150 g/kg; mineral mixture, 20 g/kg; common salt, 10 g/kg*.

### Methodology for Measuring Residual Feed Intake (RFI) in Buffalo Calves

The BWs of individual animals, recorded at the time of initiation and completion of the trial, were compared to determine the average daily weight gain (ADG):

$$\begin{array}{l} \text{Average Daily Gain}=\text{Total weight gain during trial}\\ \;÷\text{No}.\text{ of days} \end{array}$$

Daily feed intake was recorded for each animal, and body weight was taken fortnightly. The average DMI for the 112-day feeding period was regressed on average metabolic body weight (BW 0.75) and average daily gain (ADG) (30). RFI was computed for each animal and was assumed to represent the residuals from a multiple-regression model regressing dry matter intake (DMI) on ADG and average metabolic BW (MBW) (BW 0.75). The actual DMI minus the predicted DMI corresponds to the RFI. The base model used was Yj = β0 + β1MBWj + β2ADGj + ej, where Yj is the DMI of the jth animal, β0 is the regression intercept, β1 is the regression coefficient on MBW, β2 is the regression coefficient on ADG, and ej is the uncontrolled error of the jth animal (RFI). A more efficient animal has a negative RFI (observed feed intake is less than predicted feed intake), and a less efficient animal has a positive RFI (observed feed intake is greater than predicted feed intake). The allocation of animals over the two subgroups of low and high conversant was based on estimated RFI. High and low feed conversant animals were identified based on residual feed intake (RFI) as a measure FCE. The relation between blood analytes and feed efficiency in terms of RFI assigned to individual heifer was established. The study hypothesized low dry matter intake, translated into low residual energy intake, as indices of high energy conversant and so the productivity of an animal.

### Sampling

Blood samples (10 mL) were collected at each instance of initiating the trial (day 0), followed by days 30, 60, and 90 of the 96-day feeding trial at h 9.00 from 42 growing heifers in the study during July to October from the jugular vein in a serum clot-activated vacutainer (VACUETTE®). After collection, samples were centrifuged at 3,000 rpm, 4°C for 15 min. Serum was separated and stored at −20° C until analyzed. Blood serum estimates of urea, total protein, albumin, cholesterol, low-density lipoprotein (LDL), high-density lipoprotein (HDL), triglycerides, lactate dehydrogenase, serum glutamate oxaloacetate transaminase (SGOT), serum glutamate pyruvate transaminase (SGPT), and phosphorus were computed using an automated biochemical analyzer (Coralyzer 200, Tulips Diagnostics, India) and commercial kits (Coral Clinical Systems, India). Serum insulin-like growth factor-1 (IGF1), triiodothyronine (T_3_), & thyroxine (T_4_) levels were estimated using ELISA kits (Sincere Biotech Co., Ltd. Beijing). The intra-assay and inter-assay coefficients of variation were ≤9% and ≤15%, respectively.

### Blood Parameter Dataset

Blood parameters were measured in all the samples collected from 42 heifers at four different intervals, i.e., at start of the trial (day 0), followed by three more collections on the 30th, 60th, and 90th days of the feeding trial. Day-wise outlier detection for every blood parameter was applied using the box and whisker plot method in R statistical language (31), picking 32 observations out of 42. In box and whisker plots, the central mark is the median (q2); the edges of the box were the 25th q_1_ and 75th q_3_ percentiles. Points were drawn as outliers if they were larger than q_3_+W (q_3_-q_1_) or smaller than q_1_-W (q_3_-q_1_), where *W* = 1.5 (three states of vectors q1, q2, q3). Means over 4 intervals of each of the blood parameters were employed including the values of outliers imputed by the Markov chain Monte Carlo (MCMC) method (32) for 32 out of 42 animals in trial, with their corresponding age and ADG to compute the potential of feed utilization function in altering intermediary metabolic differences of high and low feed conversant based on the RFI (−0.437 to 0.359) determined in this study. The difference in average DM intake between the heifers of two energy-utilizing subgroups was recorded as 100 g per day. The av. body weight (BW) gain over 42 heifers was 45 kg during the feed trial with an average initial BW of 155 kg ranging between 96 and 214, attaining final BW as 200 kg (ranging between 147 and 254 kg). The average daily weight gain (ADG) remained 590 g/day, ranging between 382 and 807 g/day. Ten animals were selected in each of the high and low feed conversant subgroups, based on daily DMI (between 3.3 and 6.0 kg) to analyze the variation in blood attributes over two feed utilizing levels in heifers.

### Machine Learning Platform

All the machine learning algorithms were implemented using the Java-based Waikato Environment for Knowledge Analysis (WEKA) data mining software package (33) available as an open source. Machine learning models can account for complexity of predictor and response variable relationship over correlation analysis, infested with the limitation of determining only linear relationship positive, negative, or none between variables, without showing causation. An individual heifer with a set of 14 blood parameters and estimated value of RFI (FCE) was described as the response variable.

**SMO reg** uses support vector machines for regression (34). **IBK** assigns an outcome value as the nearest neighbor-based algorithm, by taking the average of the numerical target of the K nearest neighbor (35). **Locally Weighted Learning (LWL)** algorithm uses an instance-based algorithm to assign instance weights, which are then used by a base classifier for prediction (35). **Random Forest (RF)** is an ensemble learning algorithm consisting of a number of individual decision trees. At each node of the decision tree, a bootstrapped sample of training instances is evaluated along with a random subset of features followed by combination of decision outcome of individual decision trees 23 (36).

**Isotonic Regression** is a repressor for a dataset of low-level oscillations (noise), enabling capture of the internal dynamics contrary to obtaining false high scores by considering the slope as a straight line in linear regression. It minimizes the function

$$\begin{array}{l} f\left(x\right)=\overset{n}{\underset{i=1}{\sum }}W_{i\;}{\left(Y_{i\;}-{Ŷ}_{i}\right)}^{2} \end{array}$$

where Y_i_ = y_1_, y_2_,……., y_n_ are observed responses and _1,2_,……, _n_ are the unknown response values and W_i_ are the positive weights, fit for the least square method for monotonically increasing/decreasing functions (35).

**Additive Regression** has been used to generate accurate regression (37, 38) at each iteration, the residuals left over as a meta-classifier in the preceding iteration, to fit the model.

**Partial Least Square Regression (PLSR)** (39, 40) is a multivariate statistical procedure to build explanatory and predictive models to analyze multiple-response (dependent) and multiple explanatory (independent) variables, where high multicollinearity in small sample size ceases reliable conclusions due to classical regression solution. The algorithm was applied using XL stat (trial version).

### Performance Evaluation Parameters

Performance of the machine learning algorithms was evaluated using 10-fold cross-validation and leave-one-out cross-validation methods. The dataset was divided into ten equal divisions in 10-fold cross-validation, where 9 divisions are used for training and the one left division is used for testing. This process is repeated till each fold is used once for testing. Leave-one-out cross-validation (LOOCV) is a special case of K-fold cross-validation, where each sample is used once for testing. LOOCV is considered to be the most objective test and is preferred for small data-set instances (41–46). The performances of the machine learning algorithms are further evaluated using performance evaluation metrics—correlation coefficient, mean absolute error, and root mean square error.

### Mean Absolute Error (MAE)

The mean absolute error (MAE) is defined as the difference between values predicted by a model and the values actually observed from the real environment. It is derived from the unaltered magnitude (absolute value) of each difference

$$\begin{array}{l} \text{MAE}=\frac{\sum_{i=1}^{n}|X_{obs,i}-X_{model,i}|}{n}\\ \text{ Where},\text{ n}=\text{the number of samples}\\ \;{\text{X}}_{\text{obs},\text{i}}=\text{observed value of FCE}\\ \;{\text{X}}_{\text{model},\text{i}}=\text{predicted value of FCE} \end{array}$$

**Root Mean Square Error (RMSE)** is also known as the root mean square deviation, calculated as the difference between the values predicted by a model and the values actually observed from the real environment (FCE) that is being modeled.

$$\begin{array}{l} \text{RMSE}=\sqrt{\frac{\sum_{i=1}^{n}{\left(X_{obs,i}-X_{model,i}\right)}^{2}}{n}}\\ \;{\text{X}}_{\text{obs},\text{i}}=\text{observed value of FCE}\\ \;{\text{X}}_{\text{model},\text{i}}=\text{predicted value of FCE} \end{array}$$

### Model Quality Indices for PLSR

The quality of the PLSR model was evaluated by the three model quality indices, i.e., Q^2^ cumulated (Q^2^ cum), R^2^Y cumulated (R^2^Y cum), and R^2^X cumulated (R^2^X cum). Q^2^ cumulative gives global goodness of fit and the predictive accuracy of the first components.

$$\begin{array}{l} Q^{2}cum\left(h\right)=1-\overset{h}{\underset{j=1}{\prod }}\frac{\sum_{k=1}^{N}PRESS_{kj}}{\sum_{k=1}^{N}SSE_{k\left(j-1\right)}} \end{array}$$

The index involves the calculation of PRESS statistic (using cross-validation) and the sum of squares of errors (SSE) with one less component. R^2^Y cum gives the correlation between the explanatory (independent) variables with the components and R^2^X cum correspond to the correlations between the dependent variables with the components.

## Results and Discussion

The objective of the present study was to have an insight into physiological dynamics involving “pattern change” in various blood parameters in respect of average daily weight gain (ADG) and feed conversion efficiency (FCE) depicted as residual feed intake (RFI). The latter was estimated as the difference of actual and predicted DM intake (DMI) for each individual animal. Variation over mean BW, ADG, DMI, and residual feed intake was determined in heifers.

### Blood Attributes

Repeated-measure ANOVA estimates of all the blood parameters covered under the study are depicted in Table 2. Levels of total protein, triglycerides, SGOT, and phosphorus in blood serum are comparable with earlier reports (47). The level of albumin and cholesterol in serum of heifers was estimated to be lower, but LDH and SGPT levels were higher than corresponding values reported in adult buffaloes. Higher energy status of heifers than adult buffaloes corroborates with higher energy status during active growth. Significant individual variation was recorded in respect of blood thyroxin, a growth regulator in these animals.

**Table 2:** Descriptive statistics of all blood parameters, ADG along with FCE (*n* = 32).

**Blood parameters**	**Minimum**	**Maximum**	**Mean**	**Std. deviation**	**Individual variation (*F*-value, *P* ≤ 0.05)**
Urea (mg/dl)	14.400	29.250	22.861	2.653	0.31, 1.000
Total Protein (g/dl)	4.725	6.700	5.705	0.506	0.89, 0.638
Albumin (g/dl)	1.575	2.550	2.026	0.208	0.73, 0.837
Cholesterol (mg/dl)	53.000	80.725	63.706	7.026	0.85, 0.684
LDL (mg/dl)	17.575	43.825	30.643	5.657	0.99, 0.488
HDL (mg/dl)	42.350	61.675	51.931	4.723	0.35, 0.999
TG (mg/dl)	31.900	46.200	38.605	3.110	0.78, 0.780
LDH (U/l)	676.400	1221.725	827.352	105.570	0.20, 1.000
SGOT (U/l)	52.250	87.850	66.127	8.183	0.22, 1.000
SGPT (U/l)	35.175	50.100	42.125	3.504	0.15, 1.000
Phosphorus (mg/dl)	5.100	6.700	5.875	0.408	0.62, 0.934
IGF-1 (ng/ml)	96.535	183.234	127.335	20.232	0.40, 0.997
T-3 (ng/ml)	1.432	1.952	1.639	0.128	0.46, 0.990
T-4 (ng/ml)	44.597	160.357	103.794	26.210	1.69, 0.029^*^
ADG (kg)	0.369	0.729	0.549	0.097	
FCE (RFI)	−0.204	0.359	0.008	0.133	

* *Significant at p < 0.05*.

### Variation in Levels of Blood Attributes and Their Test of Significance Over Feed Utilizing Efficiency

The two-sample *t*-test was carried out on estimated mean values of each of the blood attributes to record the variation in circulatory levels in respect of the difference in two subgroups, each having ten animals bearing extremely high or low feed conversion efficiency, i.e., residual feed intake (RFI). Equality of the estimated means was derived from samples collected on the initial day (day 0) followed by the 30th, 60th, and 90th days of the feeding trial, for every blood attribute tested in the study by the two-sample *t*-test, comparing two categories of high and low feed utilization efficiency animals (Table 3). Blood urea and SGPT levels differed in animals of high and low feed conversion efficiency subgroups initially at the time of initiating the trial, which was recorded non-significant later during the trial, indicating the uniform dietary status of study animals under institute management during the trial. However, total serum protein differed between animals of two feed efficiencies significantly on day 1 (<0.05) and day 90 (<0.001) of the trial, indicative of different pathways of protein utilization for the same productivity in two subgroups of heifers. While comparing the serum level of blood attributes between low and high feed conversant heifers, significant elevation was recorded, respectively, in albumin 1.8/2.1 (*p* < 0.05); cholesterol, 47.9/60.8 (*p* < 0.001); LDH, 534/721 (*p* < 0.05); SGOT, 35.7/46.8 (*p* < 0.001); T_3_, 1.5/1.8 (*p* < 0.05) on day 90; and T4 (*p* < 0.001) on day 30 of the feeding trial.

**Table 3:** Test of significance (computed t stat values) of blood metabolite(s) over different feed utilizing heifers (*n* = 20).

**Period during feed trial**	**Zero d**	**30 d**	**60 d**	**90 d**
Urea (mg/dl)	0.315	2.078^*^	1.573	1.547
Total protein (g/dl)	2.171^*^	0.886	0.619	4.596^**^
Albumin (g/dl)	0.698	0.211	1.582	2.028^*^
Cholesterol (mg/dl)	0.265	0.455	1.938^*^	3.182^**^
LDL (mg/dl)	0.650	0.245	1.191	0.196
HDL (mg/dl)	0.279	1.358	1.136	4.444^**^
TG (mg/dl)	0.709	0.305	3.152^**^	0.775
LDH (U/l)	0.341	0.131	2.232^*^	1.833^*^
SGOT (U/l)	1.344	0.985	2.098^*^	3.123^**^
SGPT (U/l)	2.995^**^	0.925	1.4631	1.506
Phosphorus (mg/dl)	1.702^*^	0.810	1.857	1.072
IGF-1 (ng/ml)	0.734	0.146	2.449^**^	0.111
T-3 (ng/ml)	1.096	2.203^*^	0.300	−0.377
T-4 (ng/ml)	0.607	2.454^**^	0.775	0.775

* *Significant at (p > 0.05)*.

** *Significant at (p > 0.001)*.

*Bold values indicates the significant*.

Total protein was found significantly higher (<0.05) in animals of the higher-efficiency subgroup. Insulin is known to diverge from IGF-I along with growth hormone (GH), where the function of these hormones is known to link the regulation of both nutrient availability and its repletion, continuing to provide adequate signals and substrate for growth (48). Pro-insulin and IGF1 modulate carbohydrate metabolism, which stimulates glucose transport and inhibits insulin sensitivity. Low IGF I estimated in efficient conversant is found to be associated with metabolic deviations related to lower cholesterol at day 60 (*p* < 0.05) and day 90 (*p* < 0.001) and lower triglycerides on day 60 (*p* < 0.001) during the feed trial in the present study (Table 2) instead of hyperlipidemia and hyperinsulinemia reported in other studies (49). A higher level of IGF1 in the subgroup of less efficient energy-utilizing animals indicates a higher stimulus to body for making metabolic changes for growth; however, secretion of higher IGF1 in circulation might also suggest inhibitory feedback influence on the GH/pituitary axis, thus affecting feed utilization efficiency. Significantly low (*p* < 0.001) SGPT (59.5 ± 0.64 U/L) was recorded in less efficient animals compared to efficient animals having a higher level of 67.72 ± 0.78 U/L, which corroborates with other reports in cattle (7, 24), further indicating gluconeogenesis as the preferred energy pathway in efficient animals. A significant (*p* < 0.05) difference in serum urea level of less efficient vs. highly efficient animals, i.e., 23.83 ± 0.35 vs. 20.94 ± 0.51 mg/dl (on day 30 of trial), indicates the effect of change in season during this particular period of July to August months covered during the trial in the present study. Onset of rains in the month of August may influence the dietary patterns in animals along with climate change. Also, downregulation of different transaminases with corresponding lowering in serum urea levels was reported by other researchers (24) in cattle. Contrary to earlier studies (7) performed in beef cattle, serum SGOT levels were recorded to be higher in efficient buffalo heifers than in the inefficient subgroup of animals in the present study. The difference between the species in respect of SGOT levels in two feed efficiency subgroups of buffalo heifers may be attributed to the difference in rumen microbiota and functioning of liver of both species (50). Efficient heifer calves also tended to have a lower concentration of T_3_ during the performance evaluation (*p* < 0.05), compared to the efficient heifer calves as reported earlier (23). It is also documented that during growth, T3 has a synergistic relationship with the growth hormone in heifers (51), supporting the argument of metabolic rate differences between heifer calves of distinct feed efficiency classifications.

### Relationship of Blood Parameters and Average Daily Gain With Feed Conversion Efficiency

The study of the interaction of the blood parameter in relation to FCE may facilitate understanding of intricate dynamics of intermediary metabolism during growth. A vast variation in physiological levels of blood attributes was observed in heifers. The correlation matrix (Table 4) depicts a linear relationship of blood attributes and average daily weight gain [ADG] with feed conversion efficiency [FCE]. Total protein and albumin were observed to have a significant positive correlation (*p* < 0.05) with FCE, while albumin was correlated only with ADG. Triiodothyronin (T_3_), a growth moderator, and thyroxin (T_4_) showed a negative correlation with ADG, with its optimum level in circulation in efficiently feed-utilizing heifers, which corroborates with an earlier study (10). A significant positive correlation of LDH and SGOT with FCE indicates that feed utilization is an energy-dependent function, which requires higher reducing power for optimum productivity of the animal. IGF 1 and its interaction with the other blood parameters were observed to be highly influential in high FCE animals, corroborating with earlier findings (52).

**Table 4:** Correlation matrix between the different blood parameters, ADG and FCE.

**Variables**	**Urea**	**Total protein**	**Albumin**	**Cholesterol**	**LDL**	**HDL**	**TG**	**LDH**	**SGOT**	**SGPT**	**Phosphorus**	**IGF-1**	**T-3**	**T-4**	**ADG**	**FCE**
Urea	**1**	0.1531	−0.0535	0.0495	−0.2274	0.0761	0.0836	−0.3115	−0.0511	0.1148	0.1812	−0.0764	−0.0839	−0.1645	−0.1266	−0.0522
Total protein	0.1531	**1**	**0.6087**	**0.4754**	0.1116	−0.1849	0.3266	**0.3846**	**0.4634**	0.2670	−0.1321	0.0274	−0.1402	−0.3176	−0.0701	**0.4726**
Albumin	−0.0535	**0.6087**	**1**	0.3386	0.0153	−0.0692	0.3302	**0.4834**	**0.3815**	0.2246	0.0302	0.1523	−0.1492	**−0.4111**	**0.3610**	**0.3845**
Cholesterol	0.0495	**0.4754**	0.3386	**1**	0.2852	0.0164	0.2571	0.2837	**0.4533**	0.2687	0.0352	0.0176	−0.1116	**−0.3830**	0.2518	0.2035
LDL	−0.2274	0.1116	0.0153	0.2852	**1**	0.2516	**−0.3822**	−0.0740	0.0114	−0.0972	−0.1579	**0.3600**	−0.0788	0.0475	−0.0089	−0.1762
HDL	0.0761	−0.1849	−0.0692	0.0164	0.2516	**1**	0.2171	−0.2663	0.0140	0.1140	−0.1671	**0.4216**	−0.2664	0.1514	0.0718	−0.2444
TG	0.0836	0.3266	0.3302	0.2571	**−0.3822**	0.2171	**1**	0.2726	**0.3947**	0.3329	0.1378	−0.1410	−0.0374	−0.1962	−0.0223	0.2621
LDH	−0.3115	**0.3846**	**0.4834**	0.2837	−0.0740	−0.2663	0.2726	**1**	**0.4422**	0.2288	−0.2182	−0.0071	−0.0240	−0.3494	0.0221	**0.3938**
SGOT	−0.0511	**0.4634**	**0.3815**	**0.4533**	0.0114	0.0140	**0.3947**	**0.4422**	**1**	**0.7233**	−0.2136	0.2169	0.0470	**−0.3627**	0.0287	**0.4042**
SGPT	0.1148	0.2670	0.2246	0.2687	−0.0972	0.1140	0.3329	0.2288	**0.7233**	**1**	−0.0494	**0.3979**	−0.0843	−0.2316	0.2218	0.1553
Phosphorus	0.1812	−0.1321	0.0302	0.0352	−0.1579	−0.1671	0.1378	−0.2182	−0.2136	−0.0494	**1**	0.0018	0.0786	−0.1030	0.0526	−0.1900
IGF-1	−0.0764	0.0274	0.1523	0.0176	**0.3600**	**0.4216**	−0.1410	−0.0071	0.2169	**0.3979**	0.0018	**1**	−0.2198	−0.1561	0.1430	0.0675
T-3	−0.0839	−0.1402	−0.1492	−0.1116	−0.0788	−0.2664	−0.0374	−0.0240	0.0470	−0.0843	0.0786	−0.2198	**1**	**0.4669**	**−0.4446**	0.1402
T-4	−0.1645	−0.3176	**−0.4111**	**−0.3830**	0.0475	0.1514	−0.1962	−0.3494	**−0.3627**	−0.2316	−0.1030	−0.1561	**0.4669**	**1**	−0.1755	−0.2082
ADG	−0.1266	−0.0701	**0.3610**	0.2518	−0.0089	0.0718	−0.0223	0.0221	0.0287	0.2218	0.0526	0.1430	**−0.4446**	−0.1755	**1**	−0.1810
FCE	−0.0522	**0.4726**	**0.3845**	0.2035	−0.1762	−0.2444	0.2621	**0.3938**	**0.4042**	0.1553	−0.1900	0.0675	0.1402	−0.2082	−0.1810	**1**

*Values in bold are different from 0 with a significance level alpha = 0.05*.

### Physiological Model

The objective of developing the **machine learning** model was to capture the dynamics of blood parameters and ADG in relation to FCE to establish performance evaluation matrices of the tested machine learning algorithms on 10-fold cross-validation and leave-one-out cross-validation for the feed conversion efficiency trait in heifers (Table 5).

**Table 5:** Performance evaluation matrices of machine learning algorithms developed for the prediction of FCE using blood parameters and ADG as predictor variables.

**Classifiers**	**Cross-validation**					
	**Correlation coefficient (CC)**		**Mean absolute error (MAE)**		**Root mean squared error (RMSE)**	
	10-fold	Leave one out	10-fold	Leave one out	10-fold	Leave one out
RBF network	0.31	0.48	0.009	0.09	0.12	0.117
Isotonic regression	0.58	0.55	0.089	0.09	0.10	0.115
SMO Reg (RBF kernel)	0.23	0.16	0.100	0.10	0.13	0.130
IBK	0.30	0.29	0.108	0.11	0.14	0.139
LWL	0.53	0.47	0.09	0.09	0.11	0.118
RF	0.35	0.27	0.09	0.10	0.12	0.127
Additive regression (isotonic regression)	***0.60***	***0.45***	***0.09***	***0.11***	***0.12***	***0.150***

*Bold values indicates the significant*.

Isotonic regression and LWL (locally weighted learning) performed better than all other machine learning algorithms, when blood parameters and ADG were used as predictor variables for FCE (response variable) (Table 5). The performance of isotonic regression is further increased by using it as a base classifier with a meta-classifier (additive regression).

We further ranked the blood parameter variables as per their importance in respect of predicting FCE, using the Relieff feature ranking algorithm (53). The Relieff algorithm works by assigning higher and lower weights to the different predictor variables based on their importance in predicting the response variable. The order of ranking in respect of predictor variables, i.e., blood parameters in relation to FCE, as response variable using Relieff algorithm, was urea >TP >albumin >cholesterol >5-LDL >HDL >triglyceride >LDH >SGOT >SGPT >phosphorus >IGF-1 >T3 >T4 >ADG in the present study.

**Figure 1:**
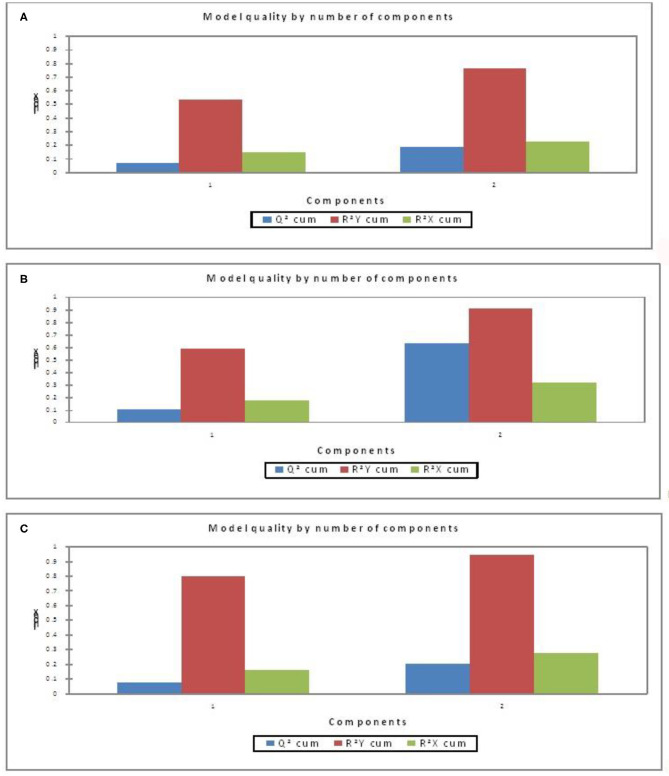
Model quality by number of components **(A)** all FCE, **(B)** higher FCE, **(C)** lower FCE).

Further, to understand the possible higher or lower interactions between the dependent and independent variables for getting insights into the possible physiological indicators of FCE, PLSR models (Figure 1) were developed as a more descriptive modeling technique. Three separate models were developed using blood parameters and ADG as the independent variable and FCE (RFI) values as the dependent variable, considering all positive and negative RFI values as model (i), only higher FCE (negative RFI) values as model (ii), and only lower FCE (positive RFI) as model (iii), respectively. Two components in the model gave better evaluation indices for the three separate models. The best PLSR model obtained was that for higher FCE (negative RFI) values (Figure 1B). The quality of the model was evaluated on the basis of Q^2^-cumulated (Q^2^ cum), R^2^Y-cumulated (R^2^Y cum), and R^2^X-cumulated (R^2^X cum) values (Figure 1); two component (new predictor variables which are constructed using the linear combinations of the original predictor variables, also known as latent variables) models are better than one-component model. The higher the Q^2^ cum, R^2^Y cum, and R^2^X cum, the better is the quality of the model.

**Figure 2:**
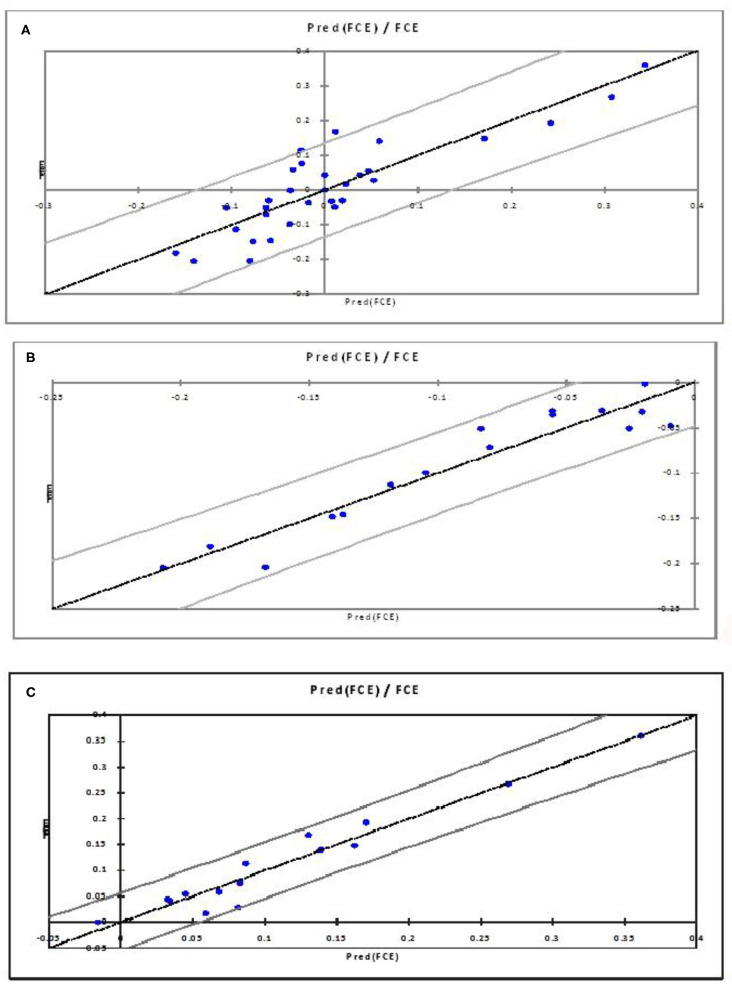
Observed and predicted FCE values for All FCE **(A)**, higher FCE/negative RFI **(B)**, and lower FCE/positive RFI **(C)**.

As per the goodness-of-fit statistic, the coefficient of determination (*R*^2^) values of the model considering all FCE values is least depicting as compared to the other two models (Table 6). The PLSR model for the higher FCE values was the best based on performance evaluation metrics (*R*^2^ **0.90680**) over lower FCE (*R*^2^ 0.9423) and all FCE (***R***^**2**^ **0.7638**) in the present study, based on the criteria of the qualifying PLSR model having *R*^2^ > 0.7 and *Q*^2^ > 0.4 (54). For all the three PLSR models, plots of observed and predicted values are shown in Figure 2. Most of the predicted values were recorded within a 95% confidence interval.

**Table 6:** Goodness-of-fit statistics for the three PLSR models.

**Goodness-of-fit statistics over different models (variable FCE)**			
**Observations**	**All FCE**	**Low FCE (positive RFI)**	**High FCE (negative RFI)**
	31.0000	16.0000	15.0000
Sum of weights	31.0000	16.0000	15.0000
DF	28.0000	13.0000	12.0000
***R***^**2**^	**0.7638**	**0.9068**	**0.9423**
Std. deviation	0.0671	0.0220	0.0262
MSE	0.0041	0.0004	0.0005
RMSE	0.0637	0.0198	0.0234

*Where, DF= the no. of degrees of freedom of the selected model*.

***R****^**2**^ = coefficient of determination, interpreted as proportion of variability of the dependent variable as explained by the model*.

*Bold values indicates the significant*.

This study indicates that variation in blood parameters and metabolic characteristics, if it does not lead to a more efficient and early selection, at least would be useful for selective genotyping for RFI/FCE through identified physiological and genetic markers.

**Figure 3:**
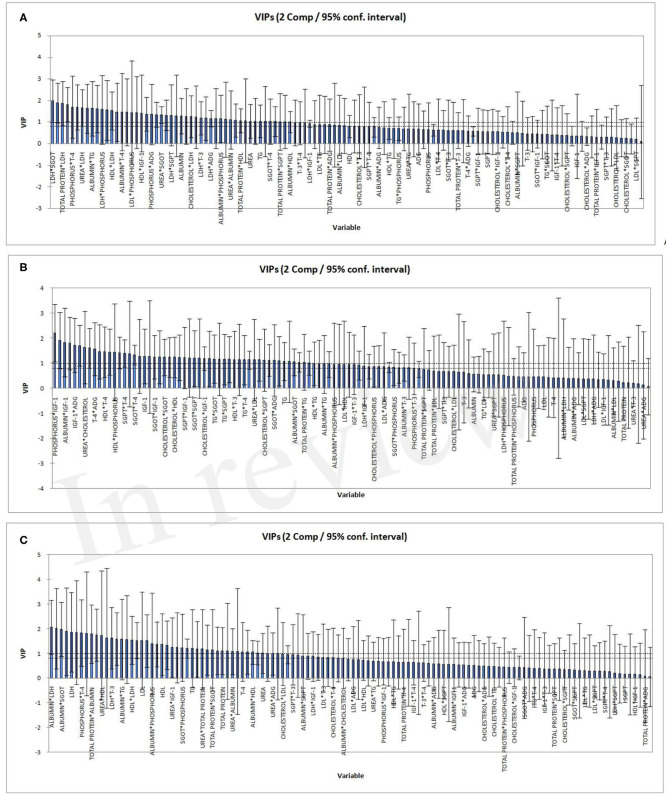
Variable importance for the projection (VIP) for the two components for the three PLSR models: all FCE **(A)**; high FCE **(B)**; low FCE **(C)**.

The two horizontal lines on the VIP bar charts (Figure 3) represent the two thresholds at 0.8 and 1. The variables having moderate influence depicted as VIP score between >0.8 & <1. Those highly influential variables have a VIP score >1. VIP-values >0.8 are significant (55) for blood parameters and ADG which differ in respect of all the three PLSR models. In the higher FCE group, IGF1 and its interaction were highly influential, while in the lower FCE group, albumin and its interaction were more influential (Table 7). IGF1 is known to regulate the levels of blood glucose, mostly (up to 90%) by gluconeogenesis, using non-carbohydrate entities as amino acid metabolism (28, 56). This study reveals the relation between IGF1 and FCE in growing young female buffalo calves corroborating with earlier reports (57).

**Table 7:** Significant interactions among blood metabolic indicators based on VIP charts emerged from PLSR models.

**Blood attribute interactions**		
**Using all RFI**	**Negative RFI/higher FCE subgroup**	**Positive RFI/lower FCE subgroup**
LDH*SGOT	Phosphorus*IGF-1	Albumin*LDH
TP*LDH	Albumin*IGF1	Albumin*SGOT
Phosphorus*T4	IGF1*ADG	LDH
Urea*LDH	Urea*Cholesterol	Phosphorus*T4
Albumin*TG	T4*ADG	TP*Albumin
LDH*Phosphorus	HDL*T4	Urea*HDL
HDL*IFG-1	HDL*Phosphorus	LDH*T3
Phosphorus*ADG	SGPT*T4	Albumin*T4
Urea*SGOT	SGOT*T4	HDL*LDH
LDH*SGPT	IGF1	LDL
Albumin	SGOT*IGF1	Albumin*Phosphorus
Cholesterol*LDH	Cholesterol*SGOT	HDL
LDH*T3	Cholresterol*HDL	UREA*IGF1
LDH*AFG	SGPT*IGF1	SGOT*Phosphorus
Albumin*Phosphorus	SGOT*SGPT	TG
Urea*Albumin	Cholesterol*IGF1	Urea*TP
TP*HDL	TG*SGOT	TP*SGOT
Urea	TG*SGPT	TP
Triglyceride	HDL*T3	Urea*Albumin
SGOT*T4	TG*T4	T4
TP*SGPT	Urea*LDL	Albumin*HDL
Albumin*HDL	Cholesterol*SGPT	Urea
T3*T4	SGOT*ADG	Urea*ADG
	Triglyceride	
	Albumin*SGOT	
	TP*TG	
	HDL*TG	
	Albumin*TG	
	Albumin*Phosphorus	

## Conclusions

Blood parameters depicting intermediary metabolism were recorded for buffalo heifers, maintained at the Govt. Livestock farm. Their interactive influence along with ADG over FCE has been established using the machine learning approach in the present study. Blood analyses are known to reflect the status of energy metabolism and some attributes were related to feed efficiency of heifers.

We developed machine learning models using blood parameters and ADG as the predictor variable and FCE as the response variable. PLSR models were developed separately for all animals, only efficient (negative RFI), and inefficient animals (positive RFI), to facilitate understanding of blood parameter interaction with ADG and FCE. The machine learning model based on isotonic regression outperformed other machine learning algorithms used for modeling in the present study. Further, the predictive accuracy of isotonic regression was enhanced using additive regression. The developed machine learning models are found effective in predicting FCE accurately. Further, the ranking of predictor variables was evaluated to predict FCE. It may facilitate understanding of intricate dynamics of blood parameters underlying growth.

As deduced from the VIP charts of PLSR, FCE is affected by IGF1 and its interactions with other blood parameters in the higher FCE group. IGF1 regulates the blood glucose level, amino acid metabolism, and protein synthesis. IGF1 has also been found related with FCE in growing heifers.

The predictive accuracy of the machine learning models can be further increased by the inclusion of a broader range of blood parameters, which can then be used as a phenotypic marker for selection of efficient animals. To the best of our knowledge, this is the first report of modeling of blood attributes and ADG with FCE in *Bubalus bubalis*. Our study is the first to show that a machine learning predictive model based on blood tests alone can be successfully applied to predict FCE in heifers and could open up unprecedented possibilities in feed trial-based cumbersome diagnosis.

## Data Availability Statement

The datasets presented in this article are not readily available because it is a property of Indian Council of Agricultural Research, New Delhi. Requests to access the datasets should be directed to www.cirb.res.in.

## Ethics Statement

The animal study was reviewed and approved by Institute Animal Ethics Committee of CIRB Hisar.

## Author Contributions

PS: design and execution of experiment and developed the manuscript. AN: developed the machine learning models. SP: performed the experiment. JA and AB: blood analysis. DM and KC: statistical analysis. AR: manuscript. KY: data collection and handling. SB: data analysis. All authors: contributed to the article and approved the submitted version.

## Conflict of Interest

The authors declare that the research was conducted in the absence of any commercial or financial relationships that could be construed as a potential conflict of interest.
